# Highlighting the Benefits of Rehabilitation Treatments in Hip Osteoarthritis

**DOI:** 10.3390/medicina58040494

**Published:** 2022-03-30

**Authors:** Andrei-Flavius Radu, Simona Gabriela Bungau, Delia Mirela Tit, Tapan Behl, Bogdan Uivaraseanu, Mihai Florin Marcu

**Affiliations:** 1 Doctoral School of Biological and Biomedical Sciences, University of Oradea, 410087 Oradea, Romania; andreiflavius.radu@gmail.com; 2 Department of Pharmacy, Faculty of Medicine and Pharmacy, University of Oradea, 410028 Oradea, Romania; 3 Department of Pharmacology, Chitkara College of Pharmacy, Chitkara University, Rajpura 140401, Punjab, India; tapanbehl31@gmail.com; 4 Department of Surgery, Faculty of Medicine and Pharmacy, University of Oradea, 410073 Oradea, Romania; uivaraseanu_bogdan@yahoo.com; 5 Department of Psycho-Neurosciences and Recovery, Faculty of Medicine and Pharmacy, University of Oradea, 410073 Oradea, Romania; mfmihai27@yahoo.com

**Keywords:** hip osteoarthritis, non-steroidal anti-inflammatory drugs, physical therapy, rehabilitation treatment, Lequesne hip index, Tinetti test

## Abstract

*Background and objectives*: Due to its frequency and possible complications, hip arthrosis or hip osteoarthritis (hip OA) has a high social impact, its advanced stages eventually leading to irreversible lesions involving major complications or surgery. In the early stages, conservative treatment plays a key role in the prophylaxis of complications and in slowing down the degenerative process. The association between an appropriate drug therapy (DT) and a rehabilitation treatment (RT)—including individualized physical therapy (PT) and adapted occupational therapy (OT)—provides good results. Our objective was to highlight the benefits of associating RT with DT in patients with hip OA. *Materials and Methods:* An observational follow-up study was conducted between 2018–2021, which included 100 patients with hip OA divided into two groups: the study group—group A (50 subjects who complied with RT) and the control group—group B (who did not comply with RT). To evaluate them, the evolution of the Lequesne hip index (LHI), Tinetti test (TT) and the hip joint mobility: flexion (FH) and abduction (AH) were monitored before the beginning of the study (T0) and after one-year (T1) for each patient. The mean values of the parameters, the standard deviations, the frequency intervals, as well as the tests of statistical significance were calculated using the Student method (*t*-test) and χ^2^, ANOVA (Bonferroni) being used to compare the means. *Results:* Compared to the evolution of group B, improvements were observed in group A, as follows: in LHI group A (*p* = 0.023) vs. group B (*p* = 0.650); in TT group A (*p* = 0.011) vs. group B (*p* < 0.001); in FH group A (*p* = 0.001) vs. group B (*p* = 0.025); in AH group A (*p* = 0.001) vs. group B (*p* < 0.001). BMI changes were non-significant in both groups A (*p* = 0.223) and B (*p* = 0.513). Evaluating group A, the most significant improvements of the studied parameters were observed in the age group 41–50 years. *Conclusions*: The study reveals the benefits of combining RT with DT in patients with especially early-stage hip OA, aged up to 50 years old.

## 1. Introduction

Hip osteoarthritis (hip OA), a chronic, irreversible condition which represents the progressive destruction of articular cartilage, the most disabling of arthrosis due to its significant impact on patients’ quality of life (QoL), is the focal point of degenerative rheumatism in the hip joint. This condition is most common in adults over the age of 40, and its prevalence increases with age. This multi-etiological, but mono pathogenic disorder affects both sexes, with a slight predominance in females [1].

Anatomopathological lesions are the expression of bone tissue’s adaptation to the elements of excessive pressure that the joint undergoes in specific circumstances; in the early phases of the pathology, if these unfavorable conditions are suppressed, the bone returns to its normal state [2]. Symptomatology including mainly pain, morning dread, limitation of mobility, muscle hypotonia, or feeling of fatigue and gait disturbance evolves over time, becoming increasingly aggravating and potentially disabling. The patient experiences discomfort when exercising, walking, or ascending the stairs at first. The pain is perceived in the groin or buttock area and might spread toward the anterior part of the thigh. As the degeneration process proceeds, the pain becomes more intense, and it persists even while the patient is at rest [3]. Similarities between hip OA and rheumatoid arthritis have been identified (i.e., fatigue and overall weakness), but also specific differences. Unlike hip OA, which affects only one hip, rheumatoid arthritis affects both hips simultaneously and possibly other joints [4].

Abandoning regular physical activity and adopting a sedentary lifestyle are variables that disrupt the natural functioning of the musculoskeletal system, favoring the onset of arthrosis. In everyday practice, hip OA is often associated with a relative degree of obesity [5].

Depending on the X-ray data and clinical symptoms, three stages of hip OA can be differentiated [6]. In the initial stage of hip OA lasting several years, conservative non-surgical therapy is recommended, including specific drug treatment (DT), occupational therapy (OT), and physical therapy (PT) [7]. If hip OA is advanced (i.e., in the final stage of degenerative hip osteoarthritis), the only option is a surgical hip prosthesis, also known as total hip arthroplasty [8]. Depending on the associated pathologies and the age of patients, DT recommended to reduce inflammation and pain includes non-COX-selective non-steroidal anti-inflammatory drugs (NSAIDs) such as Ibuprofen, Ketoprofen, or Naproxen or COX-2 selective NSAIDs such as Celecoxib and Etoricoxib [9]. For gastric protection in patients requiring long-term NSAID treatment, anti-inflammatory—proton pump inhibitor medication combinations, such as naproxen/esomeprazole, are frequently recommended [10,11,12,13]. Moreover, replacement of estrogen deficiency in postmenopausal women with hormonal therapies decreases bone loss by reducing bone resorption, both in the early years following menopause and later in life. Estrogen replacement therapy after menopause may also protect against hip OA [14].

Rehabilitation treatment (RT), represented by OT and PT, is of great importance in patients with hip OA because it contributes both to increasing the level of functionality and independence and to improving the QoL of the patients. In patients with hip OA, improving the performance of activities of daily living (ADL) is a central goal of the OT. Achieving this goal is essential for increasing individual independence in hip OA patients [15]. PT plays an essential part in RT because, according to specialized research, the tailored PT programs can prevent the worsening of hip OA, relieve symptoms, enhanced individual independence, and improved QoL in patients with hip OA [16,17,18].

In people with mild-to-moderate knee or hip OA, aerobic and strength training is indicated as the first-line conservative therapy method. Considering relevant international guidelines for the use of exercise in hip OA patients, there have been few clinical exercise trials in this population. Moreover, hip OA has been assessed in a lower proportion than knee OA in clinical trials including combined programs. Patients with knee OA appear to benefit more from lower-limb OA programs than those with hip OA. Exercise therapy aims to improve joint stability, cardiovascular fitness, muscle strength, range of motion (ROM), in order to reduce pain and impairment [19,20].

When patients with hip OA and activity limitations, balance impairment, or gait limitations are observed and documented during the history or physical assessment of the patient, clinical guidelines recommend providing impairment-based functional, gait, and balance training, including the proper use of assistive devices (canes, crutches, walkers) [21]. 

It is recommended that patients with mild to moderate hip OA with impairments in joint mobility, flexibility, or pain receive manual therapy (i.e., thrust, no thrust, and soft tissue mobilization). In patients with mild to moderate hip OA, doses and time duration may range from 1 to 3 times per week for 6 to 12 weeks. As the patient’s hip mobility improves, clinicians should add exercises like stretching and strengthening to help the patient’s range of motion, flexibility, and strength continue to improve [22]. 

Even though it is known that exercises that focus on improving muscle strength and aerobic capacity can improve OA symptoms, the effects of physical activity need to be studied further. Patients with hip OA are thought to respond to exercise similarly to those with other chronic lower-limb pain disorders. To ensure optimal compliance, hip OA patients require specially designed and conducted exercise therapy. For further developing therapeutic exercise recommendations for hip OA, more effective, practical, and sustainable exercise routines are required [23].

Within PT for patients with hip OA, the active, passive, and passive-active mobilizations are necessary to achieve positive results in restoring hip mobility [24]. Exercises, postures, and mobilizations involving the closed kinetic chain relieve the patient’s symptoms by reducing stiffness and pain. Isometric and isotonic resistance contacts are essential for regaining muscular strength [25]. The goals of the PT program in hip OA are to reduce pain, increase stability, restore muscular strength, and improve walking coordination and balance [26]. When the arthrosis is mild to moderate, conservative, non-surgical therapy with specific medication and physical therapy (PT) are recommended [27]. If hip OA is advanced, the only option is a surgical hip prosthesis, also known as total hip arthroplasty. 

The goal of this study was to assess the effects of a combination of DT and RT, which includes both OT and PT tailored programs, in order to highlight the more efficient therapeutic option for treating hip OA with greater benefits than DT. 

## 2. Materials and Methods

### 2.1. Study Design and Patients’ Selection

This is a prospective cohort study enrolling 100 patients with a diagnosis of hip OA. The registered subjects were patients who underwent specialized therapy at the County Emergency Clinical Hospital, Oradea, Romania. All patients with an early diagnosis of hip OA, hospitalized during the study period, who accepted participation by signing informed consent, were evaluated for meeting the inclusion criteria (*n* = 115). They were selected based on subjective and objective anamnestic criteria. Inclusion criteria considered were undergoing RT, Rx-confirmed diagnosis of hip OA, patients with hip OA of more than 6 months, compliance with recommended DT during the study, compliance with baseline and final assessments of follow-up parameters. Exclusion criteria were the refusal to participate in the study, neoplasms of any kind, disabling comorbidities, mental illness, neurological diseases (i.e., Parkinson’s disease, post-stroke) that cause balance and coordination disorders, osteoporosis, any medication, or alteration of general condition for any cause leading to inability to perform weekly PT (i.e., antibiotic treatment, falls, or infections), history of chronic anti-inflammatory DT in the last 6 months. Initially, there were 115 subjects, 15 of them being excluded during the research from different reasons (not meeting the inclusion criteria, lost to follow-up, etc.). The study was conducted over three years, between 2018 and 2021, following each enrolled patient during 1 year, according to the guidelines of the Declaration of Helsinki [28], and under the approval of the Ethical Commission (registration no. 27615/07.11.2018) and the Ethical Council of the Emergency Clinical County Hospital of Oradea, Romania (registration no. 27493/06.11.2018).

For subjective reasons of medical discipline (availability, adherence, health costs, etc.), the indication of compliance with RT after hospital discharge was not embraced by all patients. Depending on this factor, the two groups of patients that resulted were as follows:

- group A—the study group, 50 subjects treated with DT (NSAIDs,) and RT;
- group B—the control group, 50 subjects treated only with DT (NSAIDs), according to their specific symptomatology.

Allocation to group A treated with DT and RT or group B treated with DT exclusively was made according to patient motivation. Willingness to participate and ability to comply with the PT schedule and specific indications for performing ADLs and financial availability to perform adaptive modifications at home and at work appropriate to OT were considered. 

The study conducted by Hamre et al. was evaluated and considered to exclude subject selection bias [29]. The natural recovery potential of hip OA was considered and patients with adjuvant therapies that have a major impact influencing the ability to comply with PT have been excluded. Thus, only patients with hip OA of more than 6 months were included in this study, as the natural recovery potential decreases with increasing disease duration. 

The initial and final assessment was carried out by a physiotherapist who neither participated in the subject selection procedure nor worked with the subjects in the physiotherapy programs.

During one-year period, each of the 100 patients in our research received a DT adapted to the specific symptomatology. Joint pain (the main symptom in hip OA) has two main characteristics (i.e., pain of mechanical type that occurs after exertion, disappears at rest and weather sensitivity, accentuated by cold and wet weather) [1].

The combination selected for this purpose was naproxen 500 mg/esomeprazole 20 mg. As far as DT is concerned, an important aspect is that, in hip OA, no medication has been shown to prevent the onset of the disease or to alleviate pathological changes [30], DT being used only symptomatically and, due to the presence of periodic pain only, DT was not required daily.

Every day, the PT program recommended in the hospital was carried out under the supervision of a physical therapist at varying intensities based on the individual physical capabilities. PT is recommended being continued at home in individuals with hip OA for the rest of their lives. 

**Figure 1:**
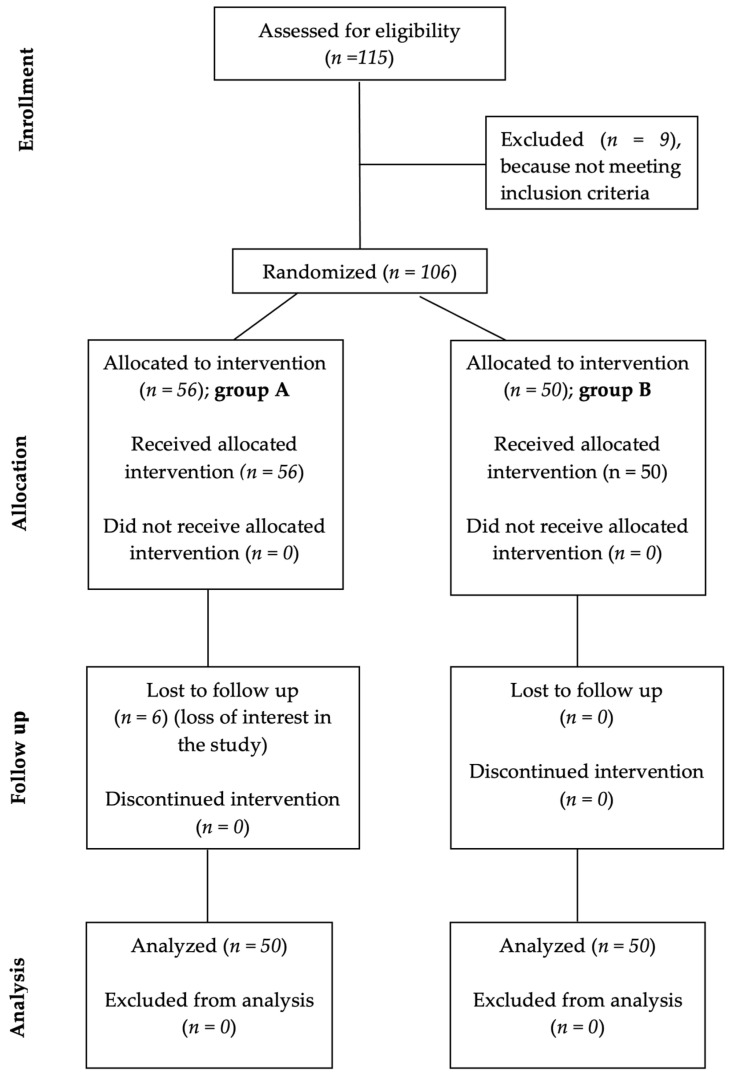
CONSORT type flow chart presenting patients’ selection and their therapeutic management.

A flow chart of the patients’ selection and their therapeutic management is depicted in Figure 1 (representing the CONSORT flowchart).

To compare the two groups, the evolution of the Lequesne hip index (LHI) [31,32,33], Tinetti test (TT) [34], and joint mobility flexion (FH) and abduction of hip (AH) were monitored. For each patient, all assessments were made before the starting the study (T0) and, at the end of the study, 12 months later (T1), by the same physiotherapist. 

LHI is a questionnaire designed to obtain information of subjective nature, from patients, about their hip affected by the disorder. The TT assessment tool measures a subject’s gait and balance. The test is scored on the subject’s ability to perform specific tasks. During the AH and FH assessment with the knee extended, carried out in the morning around 9:00 a.m., the goniometer was used to measure the active range of motion. The thigh reaches the anterior wall of the abdomen via flexion movement; the magnitude of the movement depends on the position of the knee: about 90° with the knee extended and 125° with the knee flexed. The thigh is withdrawn from the midline by the abduction action; the amplitude of the movement depends on the position of the knee: roughly 45° with the knee extended and 50–60° with it flexed [35]. The TT assessment was performed in the morning, after a short break, following the hip mobility assessment. Rested and relaxed patients were assessed in the physiotherapy room of the hospital, using walking aids as needed.

In the present study, PT, including individualized and adapted to each patient, mainly had the following objectives: increasing mobility, improving stability, and ensuring coordination and balance when walking. The PT program initially included joint mobilizations for hip joint improvement and exercises to regain stability in orthostatic position. As hip joint mobility improved, exercises to tone the muscles that anchor the hip joint were progressively started [36].

Each subject from group A followed; during a one-year period, an associated treatment between DT and RT—which was carried out in hospital over 3 weeks—included 5 sessions per week of PT and OT under the supervision of a physiotherapist. 

The exercises consisted of active and active-resistance mobilizations of hips and lower limbs. The dose and intensity of the exercises were set and progressed over time.

The basic principles followed in the PT program were painlessness (i.e., exercises were stopped if there was pain and the point at which pain occurs was not exceeded during mobilizations) and progressivity (i.e., exercises progressively increased in intensity and number of repetitions depending on the patient’s compliance). Patients were periodically monitored and according to their evolution the intensity and difficulty of the exercises were adapted.

At the end of the study, the PT program included joint mobilizations with the following working technique (i.e., active mobilization performed by the patient, followed by passive mobilization performed by the physiotherapist until the joint limit is reached, holding at that point for a few seconds and then relaxing with deep inhalation and exhalation movements). Ten repetitions were performed and then a 3-min break was taken. Exercises were performed in 2–3 series depending on the patient’s compliance on flexion, extension, and abduction movements. Another set of exercises targeted for regaining stability from orthostatism by shifting the body weight from one leg to the other and rebalancing exercises and closed kinetic chain exercises (i.e., pedaling exercises on the ergometer bike). Furthermore, muscle toning exercises target the abductor, extensor, hip joint flexor, and knee extensor muscles. 

To perform ADLs (i.e., feeding, personal hygiene, ambulation) in OT, patient education and adaptive modifications of the home and workplace are needed. The basic principle in OT is to increase QoL and achieve independence in ADL, avoiding hip joint overuse and falls. Thus, it was recommended to avoid the use of stairs, orthostasis, prolonged walking as well as avoiding vicious postures: kneeling or squatting. It was also recommended to maintain a body weight appropriate to the height and to use comfortable footwear for better stability. In the home, it was recommended to remove thresholds and slippery surfaces that can cause falls. When sitting (i.e., at the table, desk, driver’s seat), it should be considered that the hip height should exceed the knee. In the bathroom, it has been recommended to use the shower cubicle, use support bars for stability, and non-slip tiles. When shopping, it was recommended to use a trolley to protect the hip joint. Recommended activities with beneficial impact were swimming and cycling depending on the clinical condition of each individual patient [37].

The main targets of OT were to avoid the use of stairs as well as orthostasis and/or prolonged walking. OT includes exercises on the exercise bike by normal or on the back pedaling, activities that improve the ability to perform ADL, and adaptive changes at home and at work. 

All patients were recommended that RT, represented by the PT and OT program, be continued at home. They received a draft and a written description of the PT programs and OT activities and were reminded to follow them. Patient monitoring at home was done by a video call with the help of physiotherapists and student volunteers. Each month, subjects who complied with RT were once reassessed in hospital by the same physiotherapist. 

PT is an important part of the hip OA rehabilitation program since it improves the patient’s QoL. The following benefits largely justify the use of a PT program in individuals with hip osteoarthritis: better hip and hip joint mobility, improved gait, enhanced individual independence, and improved QoL [13].

### 2.2. Lequesne Hip Index and Tinetti Test 

The LHI was created to assess the severity of hip OA in drug trials in an adult French population, as well as the long-term therapy effects for hip OA and to aid in the decision-making process for hip replacement. The indicator includes symptoms of OA as well as physical functioning impairment. The instrument is currently accessible in three different formats: interview-based, self-administered, and modified versions with different scoring and wording. There are 11 questions, and the total score ranges from 0 (no pain or disability) to 24 (highest pain or maximum disability) [31,32,33]. TT is the most often mentioned assessment tool, according to medical data. The instrument has been described as the gold standard for measuring mobility dysfunctions in the elderly and a critical fall risk assessment tool in a variety of populations. Despite its widespread clinical use, multiple variations of the TT with varied scoring methods can be found, thus raising issues when reporting TT validity and reliability results [38]. 

The flexion and abduction movement were examined with the knee extended to determine hip joint mobility. The thigh reaches the anterior wall of the abdomen via flexion movement; the magnitude of the movement depends on the position of the knee: about 90° with the knee extended and 125° with the knee flexed. The thigh is withdrawn from the midline by the abduction action; the amplitude of the movement depends on the position of the knee: roughly 45° with the knee extended and 50–60° with it flexed. 

### 2.3. Statistical Analyses

Of the 115 patients who signed the informed consent form and were evaluated for inclusion in the study, 100 completed the study. To calculate the power of the test, the OpenEpi software was used and a sample size representative of 68 patients was obtained. IBM^®^ SPSS^®^ Statistics 20 software was used for statistical processing and analysis. Initially, an analysis of the data distribution was performed and, since the data distribution was normal, *p*-values were generated using a parametric test. Mean parameter values, standard deviations, frequency intervals, and tests of statistical significance were calculated by the Student method (*t*-test) and χ^2^, ANOVA (Bonferroni) being used to compare the means. The threshold for statistical significance was set at 0.05 (*p* ˂ 0.05 was considered significant, *p* ˂ 0.001 was considered highly significant, and *p* > 0.05 was considered inconsistent). The effect size (ES) was taken into account to allow the magnitude evaluation of the changes in the parameters’ values, at different times. ES values can be interpreted as follows:

- ES < 0.2—minor change;
- ES between 0.2–0.49—small change;
- ES between 0.5–0.79—moderate change and
- ES > 0.8—major change.

It is essential that ES to display the results of a quantitative study, as *p*-values identify the effect but does not show its magnitude.

Data were presented as tables and graphs/figures, and results were interpreted numerically and/or as percentages. Data processing included the following steps: description of the two groups of subjects to determine whether there were significant different characteristics, comparison of baseline values and the evolution of the studied parameters in the two groups of subjects in relation to the therapy performed, and analysis of the evolution of the subjects in group A during the study according to the group characteristics.

## 3. Results

The following parameters were analyzed: age, gender, environmental origin, body mass index (BMI), the degree of joint damage, and the parameters followed in the study to provide a valid comparison between the two groups noted as A and B (Table 1 and Table 2).

By comparing the baseline values of the parameters followed in the study, we tried to observe whether there were statistically significant differences between the two groups of subjects. Table 1 shows that there were no statistically significant differences between group A and group B at time T0:

- females are predominated in both groups, (1.17:1—group A vs. 1.27:1—group B);
- most cases (52.00%—group A vs. 50.00%—group B) were between the ages of 51–60;
- most subjects in both groups were from urban areas, and
- in terms of hip OA staging, most subjects in both groups were classified with grade 1.

By comparing the baseline values of the parameters monitored in the study, we tried to observe whether there were statistically significant differences between the two groups of subjects. Table 2 shows that the two groups with subjects included in the study did not present statistically significant difference of parameters values at T0. Table 3 compares the evolution of modifiable parameters in the two groups of subjects in relation to the therapy performed.

When comparing the two groups in terms of modifiable parameters, we have observed that, during the study, they have a different evolution. In group A, it was observed that, at time T1 compared to time T0, LHI has lower values while TT, FH, and AH have higher values. The favourable evolution of these parameters (LHI: *p* = 0.023, ES = 0.764; TT: *p*= 0.011 ES = 0.431; FH: *p* = 0.001 ES = 1.592, and AH: *p* = 0.001 ES = 1.099) is clearly influenced by the RT. Regarding the BMI evolution, a non-significant decrease is observed (T0 = 29.40 kg/m^2^ to T1= 28.30 kg/m^2^, *p* = 0.223, ES= 0.293). In group B, it was observed that, at T1 compared to T0, LHI has statistically insignificant higher values (*p* = 0.650, ES= 0.131). In contrast, TT (*p* ≤ 0.001, ES = 0.473) and AH (*p* ≤ 0.001, ES = 0.417) have significantly lower values. In addition, comparing T1 with T0, FH has statistically insignificant lower values (*p* = 0.025, ES = 0.296). Regarding the BMI evolution, in subjects from group B, a non-significant increase is observed (T0= 29.30 kg/m^2^ to T1= 29.80 kg/m^2^, *p* = 0.513, ES = 0.131).

To get a more accurate image of the impact of individualized PT programs and specific OT activities, we have assessed the evolution of the studied parameters in group A according to the age of the subjects. The patients from group A were divided into three groups according to the age of patients: 41–50, 51–60, and 61–70 years that were assessed (Table 4).

Following PT programs and OT activities, the parameters taken in the study improved significantly during the study for the age group 41–50 years (Table 5).

**Figure 2:**
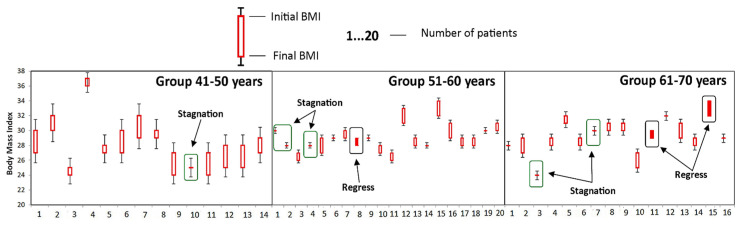
Evolution of body mass index (BMI) during the study at the group A. Stagnation—the value of the parameter obtained at time T1 is the same as the value of the parameter obtained at time T0; Regress—the parameter obtained at time T1 is less than the value of the parameter obtained at time T0.

**Figure 3:**
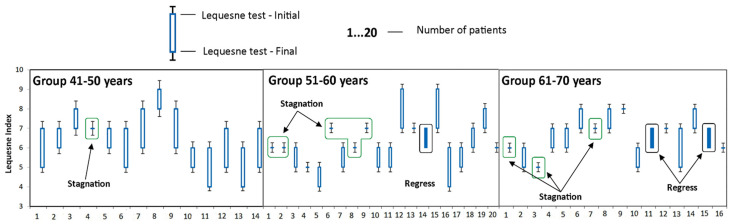
Evolution of Lequesne hip index during the study at the group A. Stagnation—the value of the parameter obtained at time T1 is the same as the value of the parameter obtained at time T0; Regress—the parameter obtained at time T1 is less than the value of the parameter obtained at time T0.

**Figure 4:**
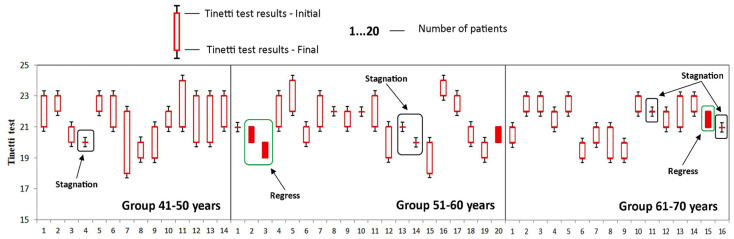
Evolution of Tinetti test during the study at the group A. Stagnation—the value of the parameter obtained at time T1 is the same as the value of the parameter obtained at time T0; Regress—the parameter obtained at time T1 is less than the value of the parameter obtained at time T0.

**Figure 5:**
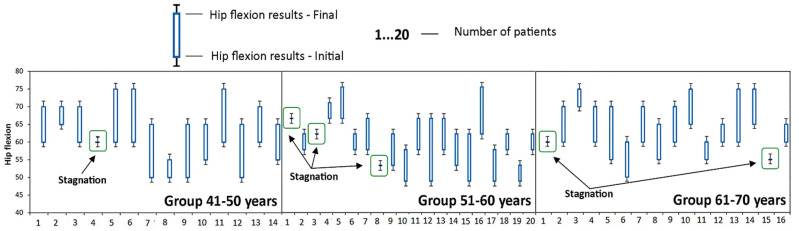
Evolution of hip flexion during the study at the group A. Stagnation—the value of the parameter obtained at time T1 is the same as the value of the parameter obtained at time T0; Regress—the parameter obtained at time T1 is less than the value of the parameter obtained at time T0.

**Figure 6:**
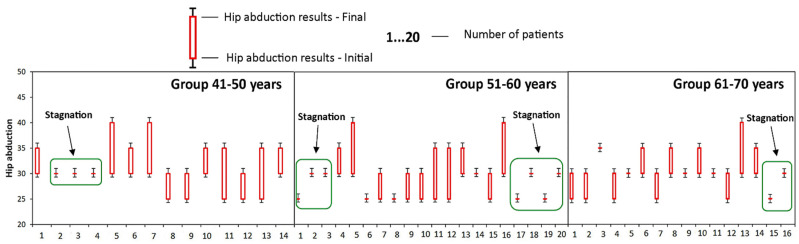
Evolution of hip abduction during the study at the group A. Stagnation—the value of the parameter obtained at time T1 is the same as the value of the parameter obtained at time T0; Regress—the parameter obtained at time T1 is less than the value of the parameter obtained at time T0.

The good evolution of patients in the 41–50 age group is also indicated by the small number of patients in whom the disease has stagnated (the value of the parameter obtained at time T1 is the same as the value of the parameter obtained at time T0), as well as the absence of those who suffered a regression of the disease (the parameter obtained at time T1 is less than the value of the parameter obtained at time T0). This was the only group where no cases of disease regression were recorded, as it can be seen in Figure 2, Figure 3, Figure 4, Figure 5 and Figure 6.

## 4. Discussion

The only option to postpone or avoid the onset of osteoarthritis is to engage in physical activity. Giving up moving and acceptance of a sedentary lifestyle are variables that disrupt the natural functioning of the musculoskeletal system, allowing arthrosis to develop [39]. Dosing and grading the physical effort are actions that can be performed within very wide limits, from the minimum effort made by passive mobilization to the mobilization with counter-resistance, which reaches close to the maximum possibilities of the subject. Intensity dosing is obtained by gradually increasing the effort and the number of repetitions, the amplitude, and the speed of movement. Dosages in complexity are made as the technique of execution is mastered. The individualization of the PT program is a basic principle, it considers the particularities related to sex, age, professional, living conditions, and environment [40]. Professional and home adaptations, components of OT, are very important in PT for patients with hip OA [41].

Subjects in group A who complied with the association of DT with RT had better hip joint mobility, better gait stability, and increased QoL demonstrated by improved symptomatology and increased independence in performing ADL.

When the two groups were analyzed using the LHI, we discovered that most of the subjects from the A group had lower scores at T1 than at T0. PT has been shown in specialized research to be effective in the treatment of hip OA, resulting in pain alleviation and increased functioning [42,43]. This aspect highlights the fact that, in general, subjects who underwent the PT program have lower LHI item values at the end of the study compared to those at the beginning of the study. However, in group B, the situation is exactly the opposite: most subjects who did not undergo PT have higher LHI item values at the end of the study than at the beginning of the study. This strongly demonstrates the benefits of PT treatment in terms of pain, maximum distance covered, and daily activities, which are the components of the LHI, as validated by other specialized research as well [16,44].

Patients with hip OA who adhered to a tailored, individualized PT program showed significant improvements in Lequesne index values. Consequently, self-esteem improved, and anti-inflammatory medication decreased [45]. 

Regarding the TT, which assesses gait balance and gait characteristics, we discovered that over 70% of the subjects in group A have recorded higher values at T1 than at T0, an aspect also highlighted by specialized research regarding the benefits of RT in terms of improving gait in patients with hip OA [46,47]. More than half of the participants in group B had lower values at T1 than at T0, explaining why, in the absence of PT treatment, patients with hip OA experience balance problems when walking as well as a change in gait quality [48]. As a result of this factor, the probability of falls with secondary complications, most notably femoral neck fractures, a major problem that severely affects QoL, increases significantly [49].

The mobility of the hip joint, as measured by FH and AH, is considerably improved in more than three quarters of the group subjects who performed regular PT, this aspect being also validated by specialized research [50,51]. In comparison, most subjects in group B showed a significant decrease in joint mobility because of not receiving PT. It was also observed that the use of NSAIDs has increased in these subjects in order to treat the symptoms of joint degeneration [24,27,52].

According to Kasnakova et al., in patients with early-stage hip OA, pain subsides following RT comprising DT and PT. Pain reduction is followed by an increase in hip joint mobility, which in turn leads to improved stability during walking [46].

One of the most common factors in our society that can lead to hip OA is obesity. From a mechanical point of view, obesity causes hip overload with increasing limitation of mobility and decreased functional independence, making walking increasingly difficult. Obesity is often associated with vascular disease and changes at the microvascular level represent the incipient events that occur in the subchondral bone in the context of hip OA. In terms of lipid metabolism disorders, the existence of a low degree of systemic inflammation and the influence of secondary proinflammatory adipokines on hip tissues create an environment that can induce cartilage, synovial membrane, and subchondral bone lesions specific to hip OA [53]. In patients with hip OA and obesity, weight loss is a major goal of the RT; this has major benefits both in terms of symptom relief and improved functionality, i.e., improved joint mobility. 

The most noticeable benefits of RT were found in patients who lost significant weight and reduced their BMI, aspect proven also by Ryan’s study [54]. By assessing patients who lost the most weight in group A, we found that they were under 50 years old. These findings are consistent with previous specialized research in the field that emphasizes the relationship between age and environment background, as well as the relevance of lifestyle [55].

In the context of younger individuals with a stronger cardiovascular reserve, a high-intensity PT can be performed. It will help both the fulfilment of PT goals and increase in energy consumption, which will also decide weight reduction [56]. This element is an additional benefit in terms of the goals of our research. According to Bennell, the benefits of physical treatment are clearer and easier to achieve in younger patients with modest anatomical alterations [57].

The main limitations of the study include the small number of participants and the relatively short time frame. Another limitation would be the lack of involvement of a dietitian in the study for nutritional recommendations to help patients lose weight. Starting from this study, it is desired to increase the volume of samples and the random selection of individuals in studies of this type, to establish the most accurate protocols in the recovery of patients with hip OA. On the other hand, we must highlight a few strengths of this research, including the design and use of a complex recovery program with an adapted, individualized physiotherapy program and specific activities for occupational therapy, as well as the evaluation of parameters that clearly underline the functional stage and symptomatic symptoms of patients. However, several randomized studies evaluating the large-scale efficacy of individualized RT in patients with hip OA are needed to support the results of this research.

## 5. Conclusions

Subjects in group A, who complied with the association of DT and RT during the study, had clear improvements in terms of LHI (pain, gait, and daily activities), TT (balance and gait characteristics), FH, and AH. The RT program advantages are obviously impacted by body weight, age, and daily activities. The clearest benefits of RT were noted in the subjects who had lost more than 10% of their weight and were <50 years old. Future research may focus on developing a strategy for both PT and OT activities in individuals with hip OA, depending on the severity of hip joint damage. Furthermore, these methods would be highly beneficial in preventing disease worsening and potentially severe consequences.

## Figures and Tables

**Table 1:** Initial characteristics of the studied groups.

Groups’ Characteristics		a.v. ± St. Dev. %	*p*
Age (years)	A	55.92 ± 6.63	0.083
	B	57.28 ± 5.32	
Gender: F/M	A	54/46	0.842
	B	56/44	
Environmental origin: U/R	A	70/30	0.501
	B	68/32	
Diagnosis: hip OA grd. 1/grd 2	A	70/30	0.830
	B	68/32	
BMI (kg/m^2^)	A	29.40 ± 4.05	0.900
	B	29.30 ± 3.86	

A—group with rehabilitation treatment, B—group without rehabilitation treatment, F—female, M—male, %—percentage values, a.v.—average value, st. dev.—standard deviation, *p* values—statistical significance, BMI—body mass index, hip OA—hip osteoarthritis, grd. 1—grade 1 of hip OA, grd. 2—grade 2 of hip OA.

**Table 2:** The initial values of parameters in the two groups.

Studied Parameters	Group A	Group B	*p*
LHI	6.70 ± 1.02	6.80 ± 0.86	0.596
TT	20.70 ± 1.18	19.84 ± 1.52	0.200
FH	58.40 ± 5.75	58.60 ± 5.29	0.107
AH	27.80 ± 2.70	27.40 ± 3.19	0.150

Group A—group with rehabilitation treatment, group B—group without rehabilitation treatment, LHI—Lequesne hip index, TT—Tinetti test, FH—flexion hip, AH—abduction hip, *p* values—statistical significance.

**Table 3:** The evolution of modifiable parameters in the two groups.

Modifiable Parameters		T0	T1	*p*	ES
LHI	A	6.70 ± 1.02	5.92 ± 1.03	0.023	0.764
	B	6.80 ± 0.86	6.96 ± 1.49	0.650	0.131
TT	A	20.70 ± 1.18	21.78 ± 3.34	0.011	0.431
	B	19.84 ± 1.52	18.52 ± 3.64	<0.001	0.473
FH	A	58.40 ± 5.75	67.20 ± 5.29	0.001	1.592
	B	58.60 ± 5.29	56.80 ± 6.76	0.025	0.296
AH	A	27.80 ± 2.70	31.80 ± 4.38	0.001	1.099
	B	27.40 ± 3.19	25.80 ± 4.38	<0.001	0.417
BMI	A	29.40 ± 4.05	28.30 ± 3.41	0.223	0.293
	B	29.30 ± 3.86	29.80 ± 3.75	0.513	0.131

Group A—group with rehabilitation treatment, group B—group without rehabilitation treatment, LHI—Lequesne hip index, TT—Tinetti test, FH—flexion hip, AH—abduction hip, BMI—body mass index, T0—start of the study, T1—end of the study, *p*-values—statistical significance, ES-effect size.

**Table 4:** Characteristics of the age groups.

Groups’ Characteristics		a.v. ± St. Dev. %	*p*
Gender: Female/Male	41–50	57/43	0.594
	51–60	50/50	
	61–70	62/38	
Environmental origin: Urban/Rural	41–50	57/43	0.852
	51–60	65/43	
	61–70	62/38	
Diagnosis: hip OA grd. 1/grd. 2	41–50	69/31	0.876
	51–60	70/31	
	61–70	44/56	
Body mass index (kg/m^2^)	41–50	29.07 ± 2.95	0.355
	51–60	29.35 ± 1.81	
	61–70	29.50 ± 2.10	

**Table 5:** The evolution of modifiable parameters the group A during the study by age criterion.

Age (Years)	T0	T1	*p*	ES
BMI				
41–50	29.07 ± 2.95	27.07 ± 3.25	0.100	0.644
51–60	29.35 ± 1.81	28.60 ± 1.54	0.167	0.466
61–70	29.50 ± 2.10	28.94 ± 2.46	0.492	0.244
TT				
41–50	20.36 ± 1.15	22.21 ± 1.25	0.001	1.540
51–60	20.75 ± 1.21	21.50 ± 1.47	0.086	0.557
61–70	20.94 ± 1.18	21.81 ± 1.11	0.039	0.759
LHI				
41–50	7.14 ± 0.86	5.64 ± 1.15	0.001	1.477
51–60	6.50 ± 1.10	5.85 ± 1.04	0.062	0.607
61–70	6.75 ± 0.93	6.31 ± 0.95	0.197	0.468
FH				
41–50	56.79 ± 5.04	67.50 ± 5.80	< 0.001	1.971
51–60	58.75 ± 6.86	66.75 ± 6.74	0.001	1.176
61–69	59.38 ± 4.79	67.50 ± 6.32	0.001	1.448
AH				
41–50	27.86 ± 2.57	33.57 ± 3.63	0.001	1.815
51–60	27.25 ± 2.55	30.75 ± 4.67	0.006	0.930
61–69	28.44 ± 3.01	31.88 ± 3.59	0.006	1.038

T0—start of the study, T1—finish of the study, *p* values—statistical significance, BMI—body mass index, LHI—Lequesne hip index, TT—Tinetti test, FH—flexion hip, AH—abduction hip, ES—effect size.

## Data Availability

Information of the patients included are available in the registers and electronic data base of the County Emergency Clinical Hospital, Oradea, Romania.
